# PM_2.5_ and Diabetes in the Japanese Population

**DOI:** 10.3390/ijerph18126653

**Published:** 2021-06-21

**Authors:** Mihye Lee, Sachiko Ohde

**Affiliations:** Center for Clinical Academia, Graduate School of Public Health, Luke’s International University, 5th Floor 3-6-2, Tsukiji, Chuo-ku, Tokyo 104-0045, Japan; saohde@luke.ac.jp

**Keywords:** diabetes mellitus, particulate matter, PM_2.5_, air pollution, Japan, Tokyo

## Abstract

Growing evidence suggests that PM_2.5_ is associated with diabetes mellitus (DM). Although DM is a major public health concern, there has not yet been a study of this association in Japan. We used health examination data from 66,885 individuals in Tokyo, Japan 2005–2019. Cox proportional hazards models were used to evaluate an association between annual exposure to PM_2.5_ and glycated hemoglobin A_1c_ (HbA_1c_), or fasting plasma glucose (FPG). An increase of 1 μg/m^3^ in the annual average of PM_2.5_ concentration was associated (HR = 1.029; 95% CI = 1.004–1.055) with an increase in diabetes (incident + prevalent). For incident DM, a greater PM_2.5_ level was associated with more DM (HR = 1.029; 95% CI, 1.003–1.055). Compared to HbA_1c_, FPG showed a stronger association with the annual exposure to PM_2.5_ (HR = 1.065; 95% CI, 1.040–1.091). We found that greater exposure to PM_2.5_ in the long-term was associated with an increased risk of diabetes, and that the magnitude of association became stronger as the exposure duration increased. Omorogieva Ojo

## 1. Introduction

Diabetes mellitus (DM) is a major public health concern in Japan. According to a national survey, about 12% of the Japanese population had diabetes in 2016 [1]. The number of diabetics has been on the rise since the first survey was conducted in 1997. The principal causes of the increase are known to be the aging population and an increase in obesity [2], yet environmental factors for diabetes have not been discussed in Japan.

It has been well established that exposure to particulate matter, with an aerodynamic diameter of 2.5 μm or less (PM_2.5_), is associated with a higher risk of cardiovascular disease (CVD) [3,4,5,6]. Researchers have suggested that PM_2.5_ induces CVD [3] via systemic inflammation. Recent evidence indicates that chronic inflammation plays a pivotal role in developing DM as well [7].

Two meta-analyses concluded that long-term exposure to PM_2.5_ is significantly associated with an elevated risk of DM [8,9]. However, due to their authors’ restriction of source studies to longitudinal cohort studies (not yet performed in Asian countries), all of the studies analyzed in these studies had been conducted only in North American or European countries. Thus, the generalizability of the results outside of those countries would be questionable. In effect, a large cohort study of African Americans provided weak evidence for the association [10], but a cross-sectional study did not find evidence for the association in an Indian population [11]. Despite the fact that Asian populations are known to have a higher prevalence of DM compared to other race/ethnicity groups [12], evidence is still lacking and the results are inconsistent in Asian countries. In particular, there have as yet not been studies in Japan examining the association between DM and PM_2.5_. Indeed, researchers conducting a multi-country study were forced to use cigarette smoking as a surrogate measure in estimating the global burden of diabetes mellitus attributable to exposure to PM_2.5_, since no such studies had been conducted in the Japanese population [13]. Taking all these considerations into account, studies on this topic are needed for the Japanese population.

We therefore evaluated the association of PM_2.5_ with diabetes mellitus in residents of Tokyo, Japan.

## 2. Method

### 2.1. Study Population

The Japanese government mandates employers to provide their employees with annual health examinations to prevent disease or to detect disease at an early stage [14]. Typically, employees are individually scheduled to visit a medical institution to check their health and obtain a consultation with a physician. Non-employees may also choose to use the community services provided by local governments for free or to be examined at a private service at their own expense. The Center for Preventive Medicine at St. Luke’s International Hospital (hereafter called the Center), located in Tokyo, is one of the institutes that provides examination services.

We used health examination data from individuals who visited the Center between 4 January 2005 and 30 March 2019. Users tend to repeatedly attend the same institute, producing multiple records per individual.

We excluded single-time visitors to ensure the visitors had follow-up data. There was no substantial difference in demographic characteristics of single visitors versus returned visitors (data not shown). We also limited the study population to residents in the Tokyo Metropolis.

Examinees were asked to sign an informed consent permitting their data to be used anonymously for research and the study was approved by the institutional review board of St. Luke’s International Hospital.

### 2.2. Exposure Assessment

The Bureau of Environment at the Tokyo Metropolitan Government began to measure atmospheric PM_2.5_ in 2000 at a single monitor. The number of monitors has steadily increased ever since, and 81 monitoring stations were in operation in 2019. The monitors are installed both at residential areas and traffic roads. The locations of monitoring stations are illustrated on the website by the Bureau of Environment, Tokyo Metropolitan Government [15]. A tapered element oscillating microbalance (TEOM) was used to measure PM_2.5_ mass [16].

We obtained monitoring station PM_2.5_ data between 1 January 2000 and 31 March 2019. We averaged the hourly readings into the daily level of PM_2.5_. Subsequently, daily PM_2.5_ data for each monitor and day were used to calculate long-term concentration of PM_2.5_. The annual averages were calculated for each date and monitor. A year was assumed to consist of only 365 days to count lagged days in an integer format. We calculated moving averages of the monitoring station data for periods of up to 10 years.

We used the postal codes of participants’ residences to assign PM_2.5_ exposure to them. The nearest ground monitor was linked to the centroid of the postal code using the Euclidian geodesic distance. We then linked this exposure data to individual participants based on examination data mainly using ArcGIS 10.5 (Esri, Inc., Redlands, CA, USA).

### 2.3. Outcome Ascertainment

About a month before their examination, individuals were sent a package containing an instruction sheet, a questionnaire about their health and life style, including questions about their personal history of diabetes and whether they are on medication. Instructions were given to examinees to fast the night before their examination.

At the Center, venous blood samples were drawn from individuals and urine samples were collected in the morning. Assays for glycated hemoglobin A_1c_ (HbA_1c_) and fasting plasma glucose (FPG) were conducted at the Central Laboratory of St. Luke’s International Hospital. An automated glycohemoglobin analyzer (HLC-723G; Tosoh, Tokyo, Japan) was used for HbA_1C_ assays according to the National Glycohemoglobin Standardization Program (NGSP).

Individuals were asked about their history of diabetes in the questionnaire. Therefore, an individual was categorized as diabetic if his/her HbA_1c_ level was 6.5% or greater without a self-reporting history of diabetes. Total diabetes was defined as having self-reported diagnosed diabetes or undiagnosed diabetes at baseline. We defined cases of prevalent diabetes as individuals reporting a history of DM in the questionnaire with a HbA_1c_ value less than 6.5% at their first visit.

With respect to FPG, an individual with a FPG level of 126 mg/dL or higher was categorized as diabetic.

### 2.4. Covariate Data

Participants answered the questionnaire before the examination day. They reported family history of diabetes (first-degree relationship or/and grandparents), smoking status (never, current, or past), and the degree of alcohol drinking (non-drinker, light drinker, or heavy drinker), daily physical activity (very active, fairly active, not very active, or inactive). Participants who drink alcohol several times per week were classified as heavy drinkers, and those who drink less than that were classified as light drinkers. On the day of the examination, participants returned the completed questionnaires, which were then processed using optical character recognition (OCR). On the same day, individual’s height and weight were measured using a digital height measuring scale to calculate body mass index (BMI) as part of the examination.

**Figure 1:**
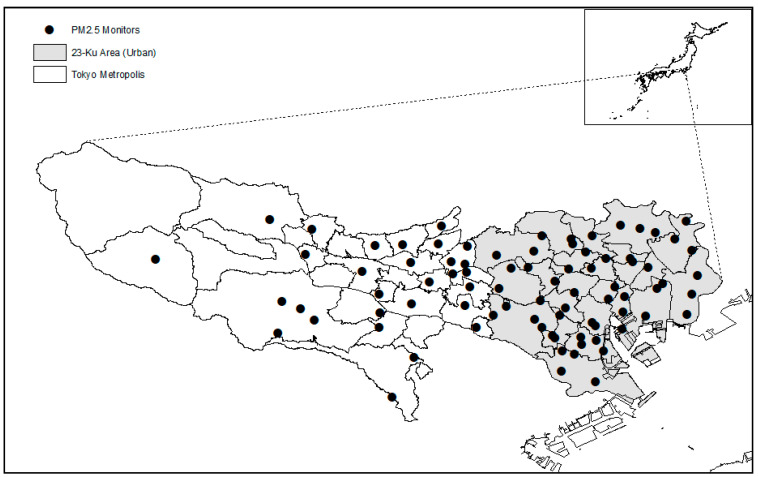
Map of administrative districts of Tokyo Metropolis and the distribution of ground monitoring stations of PM_2.5_, 2000–2019.

The Tokyo Metropolis is categorized into the following two parts: the main urban area in the east and a relatively rural area in the west (Figure 1). The eastern part corresponds to the known image of “Tokyo”, a highly urbanized area. In particular, Tokyo 23-Ku area (“*Ku*” in Japanese stands for a ward at a city level) is regarded as the urban area. Thus, the urbanization status of the postal code was determined by whether the postal code belonged to the Tokyo 23-Ku area.

### 2.5. Statistical Analysis

We applied a Cox proportional hazard model with counting process to take account of time-varying exposure and covariates. The model was stratified by age and controlled for sex, BMI, smoking status, alcohol consumption, physical activity, family history of DM, calendar year, and season. Covariates were selected if they were associated with both PM_2.5_ and diabetes or known as a risk factor for diabetes. We repeated the analysis by all (prevalent + incident) and incident DM.

We also repeated the analysis for diabetics (HbA_1c_ ≥ 6.5%) using each moving PM_2.5_ average up to 10 years. As mentioned previously, the measurement of PM_2.5_ in Tokyo began on 1 January 2000, but the period for the outcome data started 4 January 2005. Therefore, outcome and covariates data from the early in the first year (e.g., March of 2005) were not able to match with exposure data (PM_2.5_) more than 6 years since there were no exposure data available in 1999 (and before). This allowed us to utilize the entire data up to 5 years of moving average. Starting at 6 years of moving average, we had to limit outcome data from the year of 2006, and so forth.

Subgroup analyses were conducted by age group (aged ≥65 or not), obesity (BMI ≥ 25 m/kg^2^ or not), urbanization (urban or rural), and family history of DM (presence or absence) for incident diabetes.

SAS 9.4 (SAS Institute, Inc., Cary, NC, USA) was used for statistical analyses.

## 3. Results

Figure 1 delineates the map of Tokyo Metropolis and the distribution of monitoring stations for PM_2.5_. The gray area represents the Tokyo 23-Ku area (the main urban area) and the white areas are relatively less developed.

The characteristics of the 66,885 individuals are presented in Table 1. The average age of the participants was 46 years, with a standard deviation of 12 years. The number of female participants was 35,240 (52.7%). The average BMI was 22.2 kg/m^2^, with a standard deviation of 3.3 kg/m^2^. The majority of the participants lived in urban areas (89.6%). The mean concentration of PM_2.5_ was 18.5 µg/m^3^.

Table 2 shows an association of diabetic status measured by HbA_1c_ with increased annual average of PM_2.5_. An increase of 1 μg/m^3^ in the annual average of PM_2.5_ concentration was associated (HR = 1.029; 95% CI = 1.004–1.055) with an increase in all diabetes (incident + prevalent cases). The result for incident diabetes was virtually identical (HR = 1.029; 95% CI, 1.003–1.055).

Compared to HbA_1c_, FPG showed stronger associations. Incident diabetes by FPG showed the strongest association (HR = 1.069; 95% CI, 1.044–1.096).

**Figure 2:**
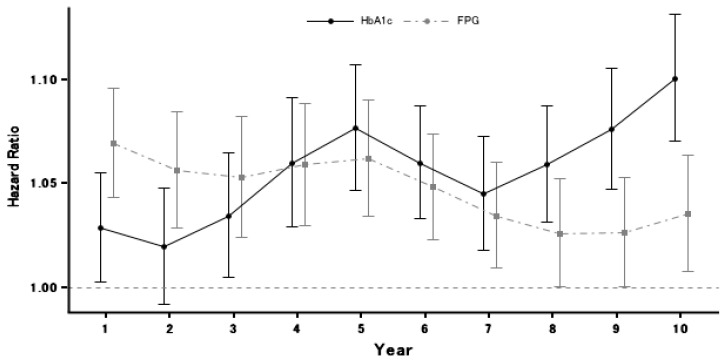
Hazard ratios for incident type 2 diabetic mellitus by diagnostic test and time window. Note: measurement of PM_2.5_ started in 2000, but the beginning of our study period was 2005; as we used a moving average over six years, we therefore restricted our outcome dataset to time points in 2006 and later.

Incident diabetes diagnosed using HbA_1c_ showed a stronger association with a longer PM_2.5_ exposure period (Figure 2). An increase of 1 μg/m^3^ in PM_2.5_ averaged over 5 year prior to check-up was associated with incident DM (HR = 1.032; 95% CI, 1.006–1.058). At any exposure duration, all of the effect estimates were greater than one, implying the proportionate relationship. On the contrary to HbA_1c_, the association of FPG with PM_2.5_ decreased over time.

Comparing the two lines in Figure 2, the magnitude of the effect estimate from FPG was greater than HbA_1c_ up to the 4th year; at this point, the direction of comparison reverses. Although both lines show a decrease in the strength of the associations between year five and year seven, after that point the strength of the association between HbA_1c_ and PM_2.5_ appears to increase, while that between FPG remains at about the same level.

**Figure 3:**
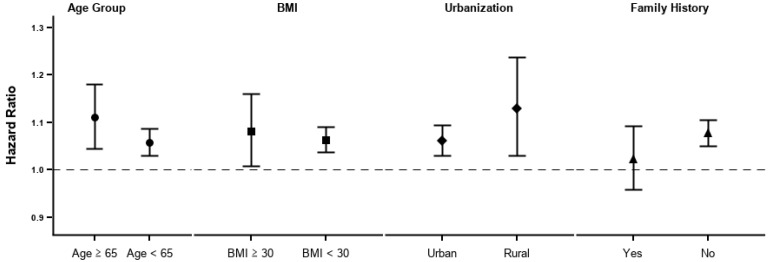
Stratified analysis by age group, obesity, residence, and family history.

The stratified analyses revealed some effect modifications; however, none of these were statistically significant (Figure 3). The old age group (aged ≥ 65) and the obese group (BMI ≥ 30) showed a stronger association than their counterparts. The rural population also showed a stronger association than did the urban population, but the confidence interval was wider than that of the urban population. Individuals with a family history of diabetes showed a lower association than those without.

## 4. Discussion

In this study, we assessed an association between long-term exposure to PM_2.5_ and diabetes mellitus in Tokyo, Japan, from 2005 to 2019. We found that a greater exposure to PM_2.5_ was associated with an increased risk for diabetes, and the magnitude of association became stronger as the exposure duration increased.

Coogan et al. found no association of PM_2.5_ with diabetes incidence among black women in the United States [10]. Researchers conducting a cross-sectional study in China reported an association between PM_2.5_ and increased diabetes prevalence, fasting glucose, and HbA1c [17]. A cross-sectional study of older adults indicated that an interquartile range increase in PM_2.5_ over one year was significantly associated with an increase in HbA_1c_ and diabetes prevalence [18]. A cohort study in Taiwan reported that long-term exposure to PM_2.5_ increases the risk of DM by 11% [19]. Meanwhile, a cross-sectional study in India observed no associations between PM_2.5_ and the prevalence of diabetes [11]. In a panel study, Brook et. al. demonstrated that PM_2.5_ was associated with reduced metabolic insulin sensitivity [20].

Our results suggest PM_2.5_ takes time to take effect on the body. We found that the longer exposure period showed a stronger effect estimate. Diabetes is a chronic disease, which develops gradually. This may explain why some previous studies did not report evidence supporting the association. Most studies used the annual PM_2.5_ level as a long-term exposure. We found that the strongest effect estimates occurred at a time point of about five years. We also observed that FPG had a stronger effect estimate at earlier time points than HbA_1c_, which is consistent with a previous study in which FPG showed a stronger association than HbA_1c_ in early exposure time windows [21]. HbA_1c_ represents the plasma glucose levels and effects thereon occurring within a few months of exposure, while FPG does the same within a much shorter time frame, e.g., a change in diet can decrease the plasma glucose level in a short period. This suggests that FPG reacts to exposures much more quickly than the HbA_1c_ test. However, HbA_1c_ is recommended by the American Diabetes Association [22] and the World Health Organization [23] because it is relatively unaffected by acute perturbations in glucose levels. It is worth noting that the measurement of PM_2.5_ began in 2000, but the beginning of the study period was in 2005, thus we limited our outcome dataset to time points beginning in 2006.

PM_2.5_ exposure may lead to diabetes via inflammation and oxidative stress [24,25]. Oxidative stress generates reactive oxygen species, which in turn induces the development of insulin resistance, β-cell dysfunction, and impaired glucose tolerance [26]. Animal studies indicated that short-term exposure to PM_2.5_ induced insulin resistance and systemic inflammation triggered by a mechanism involving oxidative stress [27,28,29,30]. Another study reported that episodic PM_2.5_ exposure is associated with systemic inflammatory responses among young and healthy non-smokers [31].

Our study has several strengths. Many studies only used self-report as the measurement of the outcome; however, we used two diagnostic tests, which worked well and have been approved by leading organizations. HbA_1c_ is a stable measurement method that has proven to work well in the Japanese population [32,33], and the HbA_1c_ test is recommended by the American Diabetes Association. FPG also forms a diagnostic test for DM. However, the American Diabetes Association has put more emphasis on HbA_1c_ than on FPG. Our use of these objective and stable biomarkers reduced information bias in the outcome measurement. Many studies have featured a cross-sectional study design. Our setting produced a pseudo-prospective study, which enabled a prospective follow-up. We, therefore, are able to exclude prevalent cases of DM and produce a less biased estimate. We also measured other variables not commonly available in other studies, such as a family history of diabetes. This allowed us to generate a better effect estimate. To the best of our knowledge, we are the first to use both HbA_1c_ and FPG in a prospective follow-up setting with a large sample size.

The limitations of our results should also be mentioned. The ecological exposure assessment of PM_2.5_ poses a limitation. Possible confounders were measured via participant self-report. Alcohol consumption and physical activity were measured by self-report using a coarse definition (e.g., low, medium, high), which may have induced some residual confounding. Data on second-hand smoking were not measured, therefore we were unable to assess its possible confounding effect. The diagnostic tests did not distinguish between type 1 and type 2 diabetes. However, considering that the study population only consists of adults and type 1 diabetes is rare in adults, the interpretation of results would be applicable for type 2 diabetes.

The reported findings may appear negligible in the clinical prospective. However, the apparent small shifts in the mean value can cause substantial change in terms of public health and entire population [34,35]. This also applies to air pollution, where exposure to air pollution is ubiquitous, and every individual is exposed to air pollution. Our study adds to the literature for public health showing an association between PM_2.5_ and diabetes.

## Figures and Tables

**Table 1:** Characteristics of the patients at baseline (*N* = 66,885).

Characteristic	Statistic (Average ± SD or *n* (%))
Age (years)	46.0 ± 12.0
Female (%)	35,240 (52.7)
BMI (kg/m^2^)	22.2 ± 3.3
Smoking Status	
Non-smoker	42,008 (62.8)
Past smoker	15,106 (22.6)
Current smoker	9771 (14.6)
Alcohol Consumption (/wk)	
Never	26,049 (38.9)
Sometimes	11,223 (16.8)
Habitual consumption	29,613 (44.3)
Daily Physical Activity	
Sedentary	8233 (12.3)
Light	22,183 (33.2)
Moderate	31,370 (46.9)
Vigorous	5099 (7.6)
Family history of DM ^a^	4036 (6.0)
Resident in Tokyo 23-Ku (urban) Area	50,424 (89.6)
PM_2.5_ (µg/m^3^)	18.5 ± 10.0

Abbreviations: SD, standard deviation; m, meter; DM, diabetes; wk, week. ^a^ Familial relationship includes first-degree family member or grandparents.

**Table 2:** Hazard ratios (95% CIs) ^a^ for diabetes mellitus associated with a 1 µg/m^3^ increase in annual ^b^ average PM_2.5_ by diagnostic test.

Diagnostic Criterion	All Diabetes	Incident Diabetes
HbA_1c_ ≥ 6.5%	1.029 (1.004, 1.055) *	1.029 (1.003, 1.055) *
FPG ≥ 126 mg/dL	1.065 (1.040, 1.091) *	1.069 (1.044 1.096) *

^a^ Stratified by age, and adjusted for sex, BMI, smoking status, alcohol consumption, physical activity, family history of diabetes mellitus, calendar year, and season. ^b^ The period of one year was defined by counting prior 365 days from the examination day not the calendar year of individual’s test date. * *p*-value < 0.05. Abbreviations: CI, confidence interval; FPG, fasting plasma glucose.

## Data Availability

The data are not publicly available due to privacy or ethical restrictions.
